# Sexually transmitted infections in male heterosexual Dutch clients who visited German cross-border female sex workers; a 3 year retrospective study

**DOI:** 10.1186/s12889-020-09240-0

**Published:** 2020-07-29

**Authors:** Carolina J. G. Kampman, Christian J. P. A. Hoebe, René Koene, Laura Kamp, Klaus Jansen, Femke D. H. Koedijk, Alma Tostmann, Jeannine L. A. Hautvast

**Affiliations:** 1 Public Health Service Twente, Postbus 1400, 7511 JM Enschede, The Netherlands; 2 Public Health Service South Limburg, Heerlen, The Netherlands; 3 Faculty of Health, Medicine and Life Sciences, Department of Medical Microbiology, Care and Public Health Research Institute Maastricht, Maastricht University Medical Center (MUMC+), Maastricht, The Netherlands; 4 Public Health Service Gelderland Zuid, Nijmegen, The Netherlands; 5 Department of Primary and Community Care, Radboud Institute for Health Sciences, Radboud University Medical Centre, Nijmegen, The Netherlands; 6 Robert Koch Institute, Berlin, Germany

**Keywords:** Female sex workers, FSW, Commercial sex, Cross border sex, Sexually transmitted infections, STI, Clients of female sex workers

## Abstract

**Background:**

Some male heterosexual clients prefer to visit a cross-border Female Sex Worker (FSW) because of cheaper sex and unsafe sex practices, and may therefore be at risk for sexually transmitted infections (STI). The objective of this study was to assess whether having commercial cross-border sex is an independent risk factor for being diagnosed with a STI.

**Methods:**

An observational retrospective study was performed using data of 8 Dutch STI clinics bordering Germany, between 2011 and 2013. All male heterosexual clients of FSWs were selected and data on country of FSW visit and occurrence of STI were used for multivariable regression analysis.

**Results:**

The study population consisted of 2664 clients of FSW. Most clients visited the Netherlands (82.4%), followed by visits to another country (beyond cross-border) (9.9%) and cross-border visits (7.8%). Clients of FSW were less likely to be STI positive when they were younger than 25 years(OR = 0.6, 95%CI 0.4 to 0.8 25–44 years and OR = 0.5, 95%CI 0.4 to 0.7 older than 45 years), and more likely when they had 20 or more sex partners in the last 6 months (OR = 2.9, 95%CI 1.9 to 4.4), did not use a condom during last sexual contact (OR = 2.2, 95%CI 1.6 to 2.9) and made cross-border visits (OR = 1.7, 95%CI 1.1 to 2.6).

**Conclusions:**

As cross-border visits appears to be a novel independent risk factor for STI in clients of FSW, this group should therefore be advised on STI prevention.

## Key points

- 2664 clients of female sex workers (FSW) were taken into analysis to determine whether making cross-border visits was a risk factor for STI.
- Most clients visited the Netherlands (82.4%), followed by visits to another country (9.9%) and cross-border visits (7.8%).
- Overall STI positivity among male heterosexual clients of an FSW was 10.4%.
- Making cross-border visits was an independent risk factor for STI positivity (OR = 1.7).
- Other independent risk factors for STI positivity were having multiple sex partners (OR = 2.9), being young (OR = 0.6, 25–44 years and OR = 0.5, > 45 years) and not using an condom (OR = 2.2).

## Background

Female sex workers (FSW) are considered a high risk group for acquiring sexually transmitted infections (STI), due to factors associated with their work and life, which makes them socially and physically more vulnerable. These include factors such as history of multiple sex partners, inconsistent condom use or co-infection with other STI and factors related to their life such as substance abuse, trauma and poverty [1, 2]. There is a potential risk for further spread of STI to the general population through their sexual contacts with male heterosexual clients with inconsistent condom use and through sex partners that are not work related. Therefore, FSW and their clients are of public health importance [3–5].

Studies to assess STI positivity in male heterosexual clients of FSW are scarce. A study from the UK among 6239 randomly selected British men assessed that 11% reported having paid for sex over a period of 5 years. This group accounted for 16% of all participating men reporting STI diagnoses (chlamydia, gonorrhoea, syphilis, HIV, genital warts, herpes, trichomonas, pubic lice, hepatitis B). Paying for sex in the previous 5 years was strongly associated with reporting higher numbers of sex partners, reporting foreign sex partners outside the UK and reporting an STI diagnosis [6]. Another British study found that men who paid for sex (MPS) were more likely to meet sex partners abroad than non-MPS (54% versus 12%) and were more likely to report having had an STI (9% of MPS, versus 3% non-MPS) [7]. A third British study demonstrated that 15% (2066/13891) of UK born HIV-positive adults acquired the HIV infection abroad [8]. These HIV infections were mostly acquired in Thailand (534), the USA (117) and South Africa (108). Those acquiring HIV infection abroad were significantly more likely to have acquired it heterosexually and to have reported sex with a commercial sex worker [8].

In the Netherlands, sex work is legal when a license is obtained. Furthermore, sex workers who work in brothels and windows are regularly inspected and visited by public health services, police and the municipality. In Germany, sex work is also legal when a license is obtained, but sex worker are not always visited by public health services, this may vary per region. Anecdotal reports from Dutch STI clinic staff suggest that clients visit cross-border FSW because of cheaper sex offered and unsafe sex practices. Therefore, the risk for STI may be higher when visiting cross border sex venues. This study aims to assess whether having cross-border sex is an independent risk factor for being diagnosed with an STI for clients of FSW living in the Netherlands. The results of this study can be used to inform sexual health clinics access policy in the Netherlands and to optimize preventive public health advice towards male heterosexual clients of cross-border FSW.

## Methods

### Study design and population

We conducted a retrospective cross-sectional study using coded data from eight STI clinics, operating at Public Health Services (PHS) in the Netherlands covering the entire Dutch-German border region. The eight STI clinic regions were included with the following main Dutch cities; Groningen, Emmen, Enschede, Arnhem, Nijmegen, Eindhoven, Venlo and Maastricht, covering an area with a population of nearly 5 million inhabitants, of 17 million (total Dutch population).

The study population was defined as all self-identified heterosexual men who attended one of the participating STI clinics in 2011, 2012 or 2013 and reported being a client of FSW in the past 6 months. Men who identified themselves as bisexual or homosexual were excluded. An FSW is defined as a woman who was indicated by the client to be an FSW, and who accepted money from the client for their sexual encounter. Clients of FSWs were defined as heterosexual men paying a woman for sex. Hereafter, we refer to these male heterosexual STI clinic clients, who reported being a client of FSW as ‘client’.

### Data analysis

For all included client records the following data were used; demographic data (age, sex, country of birth) and sexual behaviour (number of sex partners in the last 6 months (FSW-partners and non-FSW partners) and condom use during last sexual contact).

Clients were regarded STI positive when one or more STIs were diagnosed, including chlamydia, gonorrhoea, syphilis, HIV and/or infectious hepatitis B, at one or more body locations (oral, genital, or anal) at consultation. Other STI were not routinely tested, and therefore not included in analyses.

STI clinics record whether a patient is a client of a FSW, but the country where a client visits a FSW is not routinely registered. Therefore, for all included client records, open text fields were manually explored to identify whether and which country of FSW visit was entered by the STI clinic professional. Country of visit is defined as the country or countries of FSW visit, as reported by the client. A client who visits an FSW in Germany was categorized as ‘cross-border visit’; FSW visit in the Netherlands as ‘visiting the Netherlands’; FSW visits to another country than the Netherlands or cross-border, as ‘visiting another country’. As visiting numbers to other countries besides Germany and the Netherlands were low, we further combined these countries at the level of continents. If a client visited Germany and another country, they were categorized under ‘cross border visit’.

Clients who did not have a registered and named country of visit were assumed to be visiting the Netherlands. This decision was made based on the assumption that FSW visits of STI clinic patients are most likely to be within the (Dutch) region where STI care is provided and thus logically the default option and most prevalent option is actually Dutch. Therefore, we expect the blank option very likely to be the ‘Dutch’ option as STI nurses tend to omit an official registration for this common category. This assumption was confirmed when interviewing STI clinic staff on this matter. Therefore, clients with an unregistered country of visit were categorized under ‘visiting’ the Netherlands. This ‘blank’ option was the case in 80% of the registrations. We did perform a sensitivity analysis based on known country of visit to estimate the validity of our assumption that an unregistered country of visit most likely indicated a visit in the Netherlands. 20% of clients (535/2664) had a named country of visit that was entered in the open text field. Of these, most clients visited another country than the Netherlands (but not Germany) (42.8%, 229/535), followed by visits to Germany (38.7%, 207/535) and visits within the Netherlands (18.5%, 99/535). These low numbers of clients visiting the Netherlands substantiate our assumption that there is a gross under registration of clients visiting an FSW in the Netherlands.

First, descriptive statistics of demographic and sexual behavior characteristics were performed. Subsequently, numbers and percentages of being positive for any STI were described, categorized in continents. Binary logistic regression analyses were performed to identify determinants associated with STI positivity. Determinants associated with a *p* value of < 0.20 in univariable analysis were further analysed by multivariable logistic regression. STI positivity was the outcome measure and ‘country of FSW visit’ was the dependent variable. A *p* value of < 0.05 in multivariable analysis was considered to be statistically significant. Odds ratios (ORs) and 95% confidence intervals (CIs) were presented to show the association between the determinants and the outcome. Analyses were conducted using SPSS for Windows, version 25.0 (IBM Inc., Somers, New York, United States).

## Results

### Study population

As shown in Table 1, the total study population consisted of 2664 clients. Most clients visited an FSW in the Netherlands (2194/2664, 82.4%), followed by FSW visits to another country (263/2664, 9.9%) and cross-border FSW visits (207/2664, 7.8%). The continent most visited besides the Netherlands and cross-border, was Asia (122/2664, 4.6%).

**Table 1:** Characteristics of clients categorized in country of FSW visit (*n* = 2664)

	*Clients visiting the Netherlands* *N = 2194*	*Clients with cross-border visits* *N = 207*	*Clients visiting another country* *N = 263*	*All visited countries* *N = 2664*
	*N(%)*	*N(%)*	*N(%)*	*N(%)*
**Age**				
< 25 years	434 (19.8)	38 (18.4)	54 (20.5)	526 (19.7)
25–44 years	1173 (53.5)	103 (49.8)	148 (56.3)	1424 (53.5)
> 45 years	587 (26.8)	66 (31.9)	61 (23.2)	714 (26.8)
**Country of birth**				
Netherlands	1848 (84.2)	174 (84.1)	215 (81.7)	2237 (84.0)
Other	346 (15.8)	33 (15.9)	48 (18.3)	427 (16.0)
**Number of sex partners last 6 months (FSW and non-FSW)**				
0–2	780 (35.6)	58 (28.0)	92 (35.0)	930 (34.9)
3–5	788 (35.9)	67 (32.4)	95 (36.1)	950 (35.7)
6–20	379 (17.3)	55 (26.6)	49 (18.6)	483 (18.1)
> 20	147 (7.9)	24 (11.6)	23 (8.7)	221 (8.3)
Missings	73 (3.3)	3 (1.4)	4 (1.5)	80 (3.0)
**Condom used during last sexual contact**				
Yes	929 (42.3)	95 (45.9)	107 (40.7)	1131 (42.5)
No	1246 (56.8)	108 (52.2)	149 (56.7)	1503 (56.4)
Missings	19 (0.9)	4 (1.9)	7 (2.7)	30 (1.1)
**STI diagnosed at consultation**				
Yes	223 (10.2)	32 (15.5)	22 (8.4)	277 (10.4)
No	1971 (89.8)	175 (84.5)	241 (91.6)	2387 (89.6)
**Continent/country of FSW visit**^**a**^			*N(%)*	*STI positivity* *N(%)*
Other European			66 (25.1)	6 (9.1)
Asia			122 (46.4)	11 (9.0)
Africa			29 (11.0)	2 (6.9)
Oceania			1 (0.4)	0 (0.0)
North America			7 (2.7)	0 (0.0)
South America			17 (6.5)	1 (5.9)
Multiple continents			9 (3.4)	2 (22.2)

percentages may not precisely add up to 100% due to rounding

^a^Clients reporting visiting a FSW abroad, but no country of visit was reported, are not displayed (*n* = 12)

In total, 10.4% (*n* = 277/2664) of the clients were diagnosed with one or more STIs, non-stratified by country of visit. Chlamydia was the most diagnosed STI with 8.0% (214/2664) of clients being diagnosed, followed by gonorrhoea (2.4%, 63/2664), syphilis (0.3%, 9/2664), 0.2% of the clients tested positive for infectious hepatitis B (5/2664) and 0.1% of clients were diagnosed with HIV (2/2664). STI positivity was highest in clients who made cross-border FSW visits (15.5%), followed by clients who visited an FSW in the Netherlands (10.2%) and clients who visited an FSW in another country (8.4%).

### STI positivity and predictors

The predictors for STI positivity among clients are shown in Table 2. In multivariable analysis clients were more likely to be STI positive when they were younger than 25 years of age (OR = 0.6, 95%CI 0.4 to 0.8 in the age group 25–44 years and OR = 0.5, 95%CI 0.4 to 0.7 in the age group older than 45 years), had 20 or more sex partners in the last 6 months (OR = 2.9, 95%CI 1.9 to 4.4), did not use a condom during last sexual contact (OR = 2.2, 95%CI 1.6 to 2.9) and made cross-border FSW visits (OR = 1.7, 95%CI 1.1 to 2.6).

**Table 2:** Demographic and behavioral predictors for STI positivity among clients of FSW (*n* = 2664)

	Univariable regression analysis OR(95%CI)	Multivariable regression analysis OR(95%)
**Age, median years** (IQR)		
< 25 years	ref	ref
25–44 years	**0.5 (0.4–0.7)**	**0.6 (0.4–0.8)**
> 45 years	**0.5 (0.3.0.7)**	**0.5 (0.4–0.7)**
**Country of birth**		
Netherlands	ref	ns
Other	**1.3 (0.9–1.8)**	
**Number of sex partners last 6 months (FSW and non-FSW)**		
0–2	ref	ref
3–5	**1.4 (1.0–1.9)**	1.3 (0.9–1.8)
6–20	1.3 (0.9–1.9)	1.3 (0.9–1.9)
> 20	**2.9 (2.0–4.4)**	**2.9 (1.9–4.4)**
**Condom used during last sexual contact**		
Yes	ref	ref
No	**2.2 (1.6–2.9)**	**2.2 (1.6–2.9)**
**Country of FSW visit**		
Netherlands	ref	ref
Cross-border	**1.7 (1.1–2.5)**	**1.7 (1.1–2.6)**
Another country	0.8 (0.5–1.3)	0.8 (0.5–1.2)

*ref* reference, *ns* not significant

In bold: significant (*p* < 0.20 in univariable and *p* < 0.05 in multivariable analysis).

## Discussion

This is the first study assessing STI risk of clients who had sex with an FSW in another country. Study outcomes showed that the majority of clients visited FSW in the Netherlands (82,4%), followed by visits to another country (263/2664, 9.9%) and cross-border visits of FSW to Germany (207/2664, 7.8%). STI positivity was highest among clients who made cross-border FSW visits to Germany (15.5% versus 10.2% visiting the Netherlands and 8.4% visiting another country). STI risk was significantly associated with being a client with cross-border sex in Germany (OR = 1.7). Three independent risk factors for STI positivity were 20 or more sex partners in the last 6 months, being younger than 25 years and not using a condom during last sexual contact.

### Limitations

The main limitation of this study is that the country where commercial sex took place was not systematically recorded, as in the majority (80%) of records no country was mentioned. Nevertheless, we categorized clients who didn’t have a registered country of visit under ‘visiting the Netherlands’. This assumption that most of such data entries were the default option (Dutch) was in general confirmed by STI clinic staff.

Another limitation of this study is that only clients who visited a Dutch STI clinic in a region bordering Germany were selected. Therefore, the results are not generalizable to the rest of the Netherlands. Nevertheless, clients living further away from the border are less likely to visit a FSW in Germany. However, this study shows that the behavioural aspect (condom use, number of sex partners) remains of great importance in being diagnosed with an STI.

A third limitation is that it was not assessed whether condom use during the last sexual contact was with an FSW, or with another sexual contact. However, we assume that clients who reported not having used a condom during last sexual contact are clients who are more prone to or prefer to not use a condom in general.

One last limitation is that we could only include heterosexual men, based on their own identification instead of behaviour. This might have given false information, as it is known that there are men who identify themselves as heterosexual, but have sex with men as well. We do, however, think that this is a minor limitation, because the nurses at the STI clinic tend to thoroughly ask the clients about their behaviour and sexual preferences.

### Cross-border sex is an independent predictor for STI

Our study showed an overall STI positivity rate among clients of 10.4% which was comparable to other Dutch studies reporting similar proportions (9–10%) [9–13]. This indicates that our study population could be generalizable for all Dutch clients of FSW. From 2015 onwards, clients of FSW are no longer considered a high risk group by the Dutch Ministry of Health based on the balance of limited financial resources and relative low STI positivity (10% among clients of FSW versus 18% among all female? STI clinic attendees, 19% among all heterosexual men and 21% among men who have sex with men [9]). Because of this policy they are no longer eligible for free and anonymously consultations at Dutch STI clinics. Due to the relatively low STI positivity among clients of FSW, reconsidering this changed public health policy has proven to be of lesser need.

Our study showed that making cross-border visits to Germany to an FSW is an independent STI risk factor, compared to visiting the Netherlands or another country. There are other studies that show that making cross-border visits poses a higher risk of getting an STI, but these studies describe primarily high levels of STI positivity among (clients of) FSW in countries bordering Ethiopia, Vietnam and Mexico [14–17]. These countries are difficult to compare to European countries, due to different cultural context.

A possible explanation for a higher STI risk in Dutch clients, when visiting FSW in Germany as suggested by anecdotal information is the relatively higher number of Eastern European FSWs in German sex venues compared to Dutch. In an outreach study performed in Germany among ‘difficult to reach’ FSW who were not in contact with counselling and treatment institutions, 88% of the FSWs were not born in Germany. The risk of acquiring an STI was increased for FSWs born outside Germany, who work in large sex venues [18]. In a recent study performed among FSW in the Netherlands, 48% were of non-Dutch origin and 12% were of Eastern European origin [19]. Therefore, condom use is always of importance when visiting an FSW and this message should be stressed whenever clients take an STI test.

### Other predictors for STI

Our study showed that clients who did not use a condom were more often STI positive. There is a well-known relationship between condom use and reducing the risk of getting an STI [20, 21]. Though national data is difficult to generalise, due to very different national legislative, administrative and cultural contexts, there are studies that also report on the relationship between decreasing condom use by FSWs and an increasing STI positivity among FSWs [22–25]. An Italian study assessing condom use among clients of FSW showed that cumulatively, 87 and 85% of vaginal and anal intercourses were respectively reported as regularly protected by condom [26]. A Chinese study found that clients were 10 times more likely to have an STI (either self-reported or tested) than non-client Chinese men, and they were equally likely to use condoms inconsistently with their spouses [5]. Therefore, clients who prefer to make cross-border visits, should be motivated to use a condom.

A high number of sex partners (more than 20) showed to be more likely to cause a higher risk of STI which is a confirmation of many previous studies like two British studies which reported a strong association with reporting larger numbers of sex partners and STI positivity in a group that payed for sex in the past 5 years [6, 7].

### Public health implications

Working together internationally is the key in lowering STI prevalence in clients of FSW. A group of Dutch clients visit Germany because it is nearby, relatively cheap sex is offered, and unsafe sex may be practised. STI clinics in the border regions should therefore make an effort to educate clients who visit Germany and who are young, have multiple sex partners and do not use a condom on sexual risk behaviour. Also, more research is needed to assess reasons for unsafe sex practices among clients as well as FSW.

Furthermore, more effort should be made to reach FSW who are not in contact with counselling and treatment institutions [18]. Government aided educational programs in promoting condom use among FSW have also proven to be beneficial in achieving positive sexual health outcomes [27, 28]. These joined efforts could enhance wellbeing of FSW, increase knowledge among clients and could eventually lower STI prevalence in both FSW and clients of FSW.

## Conclusion

The main study finding is that Dutch clients who make cross-border visits to FSW in Germany, are more likely to be STI positive than clients who visit a FSW in the Netherlands or in another country. Furthermore, study findings showed three independent predictors; clients younger than 25 years of age, clients who have multiple sex partners (> 20 in last half year) and clients who did not use condoms were more likely to be STI positive, which might inform a targeted approach in this potential risk group. When clients of FSW meet this high risk profile, regular STI testing and eventual treatment should be considered. Furthermore, STI prevention advice should focus on clients of FSW who make cross-border visits in Germany.

## Data Availability

The datasets used and/or analysed during the current study are available from the corresponding author (CK) on reasonable request.

## Abbreviations

CI: Confidence Interval
FSW: Female Sex Worker
HIV: Human Immunodeficiency Virus
IQR: InterQuartile Range
MPS: Men who Paid for Sex
OR: Odds Ratio
PHS: Public Health Service
SPSS: Statistical Package for Social Sciences
STI: Sexually Transmitted Infections
